# Exploring the Symptoms of and Insights Into Idiopathic Opsoclonus-Myoclonus-Ataxia Syndrome in Adults

**DOI:** 10.7759/cureus.71568

**Published:** 2024-10-15

**Authors:** Ana B Santos, Anthony Hong, Isaac Hong, José D Villegas

**Affiliations:** 1 College of Medicine, University of Costa Rica, San José, CRI; 2 Neurology, Hospital San Juan de Dios, San José, CRI

**Keywords:** ataxia, autoimmune, myoclonus, opsoclonus, paraneoplastic

## Abstract

Opsoclonus-myoclonus-ataxia syndrome (OMAS) is a rare immunological central nervous system disorder that mostly affects children, and it is extremely uncommon in adults. It usually presents idiopathically, as a parainfectious condition, or as a paraneoplastic syndrome. We present a case of a patient who developed adult-onset opsoclonus-myoclonus-ataxia syndrome (OMAS) without any associated infectious or neoplastic disease, a condition that is considered very rare in Central America.

This study aimed to document a rare case of adult-onset opsoclonus-myoclonus-ataxia syndrome in a 39-year-old female, highlighting its atypical presentation and the diagnostic challenges involved. The patient presented with a one-week history of rapid-onset and progressive dizziness, nausea, and vomiting, associated with a two-day history of gait instability, memory loss, and sleep disturbances. Past medical history was only notable for psoriatic arthritis controlled with methotrexate. The neurologic examination revealed involuntary, rapid, multidirectional eye saccades consistent with opsoclonus, fast-twitching and jerking movements of the head and bilateral upper extremities consistent with myoclonus, and a wide-based gait with instability indicative of ataxia, suggesting a diagnosis of OMAS. There were no motor or sensory deficits, seizures, fever, or symptoms suggestive of infections. Brain magnetic resonance imaging and computed tomography scan of the head, neck, thorax, abdomen, and pelvis with and without contrast showed no abnormalities. Breast, abdomen, and gynecologic ultrasound, esophagogastroduodenoscopy, and colonoscopy showed no lesions suggestive of underlying neoplasia. Cerebrospinal fluid (CSF) analysis showed mild hyperproteinorrhachia and lymphocytic pleocytosis, along with oligoclonal bands. Viral, bacterial, and autoimmune encephalitis panels were negative. CSF bacterial, mycobacterial, and fungi cultures were negative. Serum viral serologies, tumor markers, and antineuronal antibodies were negative. The patient received treatment with plasmapheresis, intravenous immunoglobulin, and methylprednisolone, with significant but partial improvement of her symptoms.

## Introduction

Opsoclonus-myoclonus-ataxia syndrome (OMAS) is a rare neurologic disorder with an acute onset of opsoclonus, myoclonus, and ataxia. Although it predominantly affects children, with an incidence of 0.2 per million annually, it can also occur in adults [1]. The syndrome was first identified by Kinsbourne in 1962, who documented six cases in the pediatric population [2]. The clinical presentation of this condition can vary significantly between patients, so a comprehensive understanding of the disease is essential for accurate diagnosis. This syndrome is commonly associated with parainfectious or paraneoplastic conditions. Therefore, the diagnostic evaluation should be concentrated on ruling out these two potential causes [3].

We present a case of a patient who developed adult-onset opsoclonus-myoclonus-ataxia syndrome (OMAS) without any associated infectious or neoplastic disease, a condition that is considered very rare in Central America.

This article was previously presented as a meeting abstract at the 2024 AAN Summer Conference on July 20, 2024.

## Case presentation

A 39-year-old female patient presented to the emergency department with a one-week history of rapidly worsening dizziness, nausea, and vomiting. Additionally, she reported gait instability, memory loss, and sleep disturbances that began two days before her visit. She denied any recent history of infection, fever, seizures, headaches, or weight loss. Past medical history was remarkable for psoriatic arthritis diagnosed four years prior to this encounter and it had been treated with methotrexate since then. She did not recall any recent changes to her medication regimen. 

On the initial examination, her vital signs were within normal limits and she was oriented. Physical examination showed symmetric pupils with normal pupillary light reflex, the neck was supple, and normal reflexes and strength. She presented with involuntary, rapid, and multidirectional eye saccades, along with fast-twitching and jerking movements of the head and bilateral upper extremities. Her gait was wide-based and unstable. The remainder of the neurological and physical examination was unremarkable.

Initial laboratory tests revealed leukocytosis. Electrolytes, international normalized ratio (INR), and fibrinogen levels were all within normal limits, as detailed in Table 1. Epstein-Barr virus (EBV) IgG and Cytomegalovirus (CMV) IgG were positive, while IgM was negative for both viruses. Serologies for HIV, hepatitis C, hepatitis B, and Venereal Disease Research Laboratory (VDRL) test were all negative. Additionally, all tumor markers, including CA 15-3, CA 19-9, CA 125, carcinoembryonic antigen, and alpha-fetoprotein, were negative.

**Table 1:** Initial laboratory tests of the patient. INR: international normalized ratio

Laboratories	Result	Reference range
Hemoglobin (g/dL)	12.8	13.0-17.0
Leukocytes (cells/mm^3^)	16200	4000-10000
Neutrophils (cells/mm^3^)	12280	1800-7000
Lymphocytes (cells/mm^3^)	2430	1000-4800
INR	1.02	1.0-1.1
Fibrinogen (mg/dL)	334	200-400
Sodium (mEq/L)	141	136.0-145.0
Potassium (mEq/L)	3.5	3.5-5.1
Chloride (mEq/L)	106	98.0-107.0
Calcium (mEq/L)	8.6	8.6-10.3
Magnesium (mEq/L)	1.8	1.9-2.7
Phosphate (mEq/L)	3	2.5-4.5

A lumbar puncture was performed and cerebrospinal fluid (CSF) analysis results revealed lymphocytic pleocytosis, normal glucose, and mild hyperproteinorrhachia, as shown in Table 2, along with the presence of oligoclonal bands. India ink was negative, and VDRL was not reactive. CSF cultures, including those for bacteria, mycobacteria, fungi, and other microorganisms, were all negative.

**Table 2:** Cerebrospinal fluid analysis results and normal reference ranges.

Parameter	Result	Reference range
Appearance	Clear	Clear
Protein (mg/dL)	63	12-45
Glucose (mg/dL)	61	40-70
White blood cell count (cells/mm^3^)	14	0-5
Lymphocytes (%)	100	40-80
Neutrophils (%)	0	0-10

Molecular panels for encephalitis were negative for influenza A, SARS-CoV-2, rhinovirus, parainfluenza, and metapneumovirus. Molecular panels for meningitis were negative for *Escherichia coli *K1, *Haemophilus influenzae*, *Listeria monocytogenes*, *Neisseria meningitidis*, *Streptococcus agalactiae*, *Streptococcus pneumoniae*, *Cytomegalovirus*, Enterovirus, herpes simplex virus 1, herpes simplex virus 2, herpes simplex virus 6, human parechovirus, Varicella zoster virus, and *Cryptococcus neoformans*/*gattii*. Additionally, autoimmune encephalitis panels were also negative.

**Figure 1:**
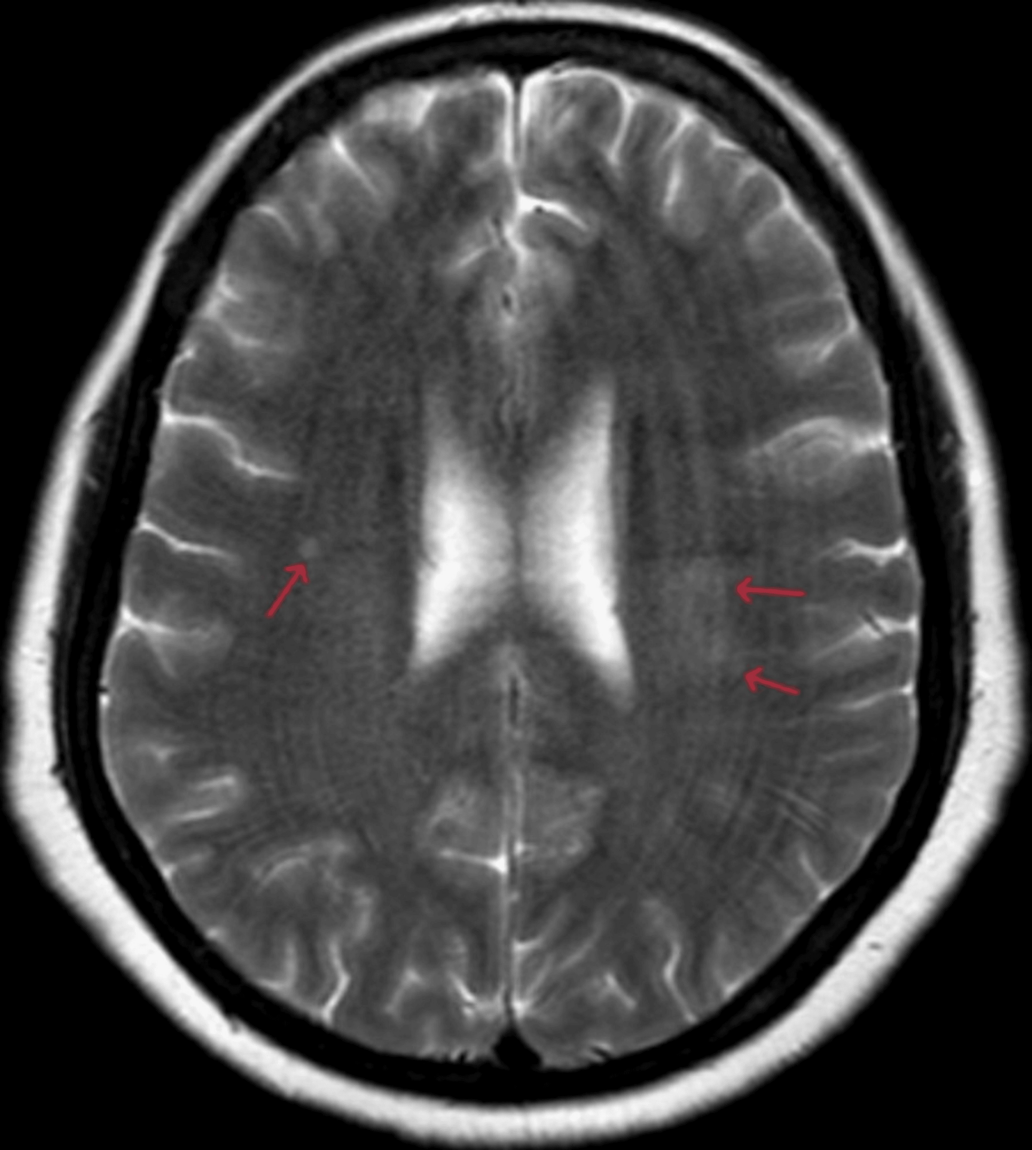
Axial T2-weighted MRI of the brain showing the lesions. Small nodular hyperintensity on the right side and diffuse hyperintense lesions on the left side (arrows).

**Figure 2:**
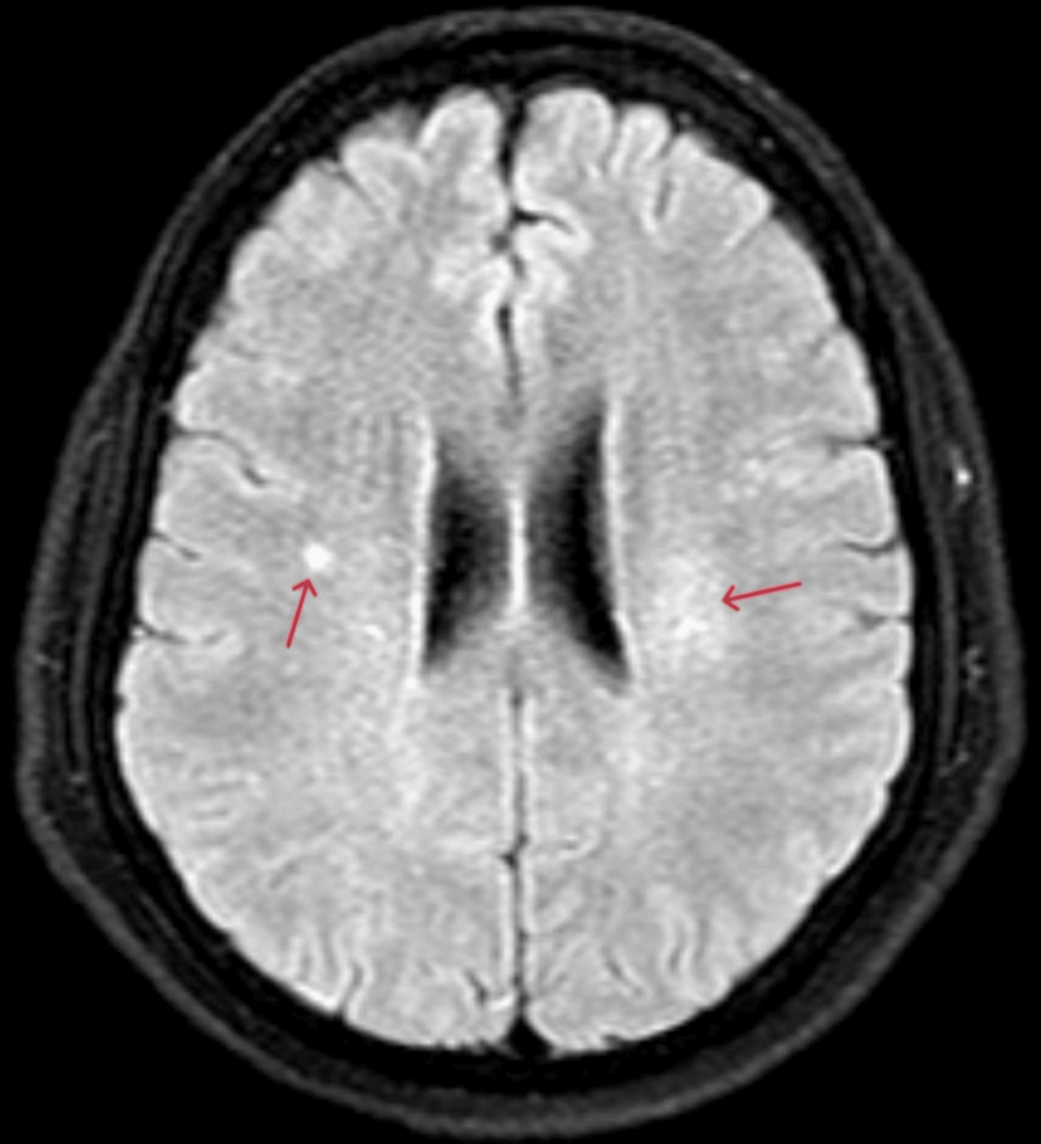
Axial FLAIR MRI of the brain showing the lesions. Small nodular hyperintensity on the right side and a diffuse hyperintense lesion on the left side (arrows). FLAIR: fluid-attenuated inversion recovery

A brain MRI revealed at least three hyperintense nodularities on T2 and fluid-attenuated inversion recovery (FLAIR) sequences, each measuring less than 3 mm, located in the right and left semioval centers (Figures 1, 2). All lesions showed enhancement with contrast. An EEG was also conducted, and the findings were within normal limits, with no evidence of epileptiform activity.

**Figure 3:**
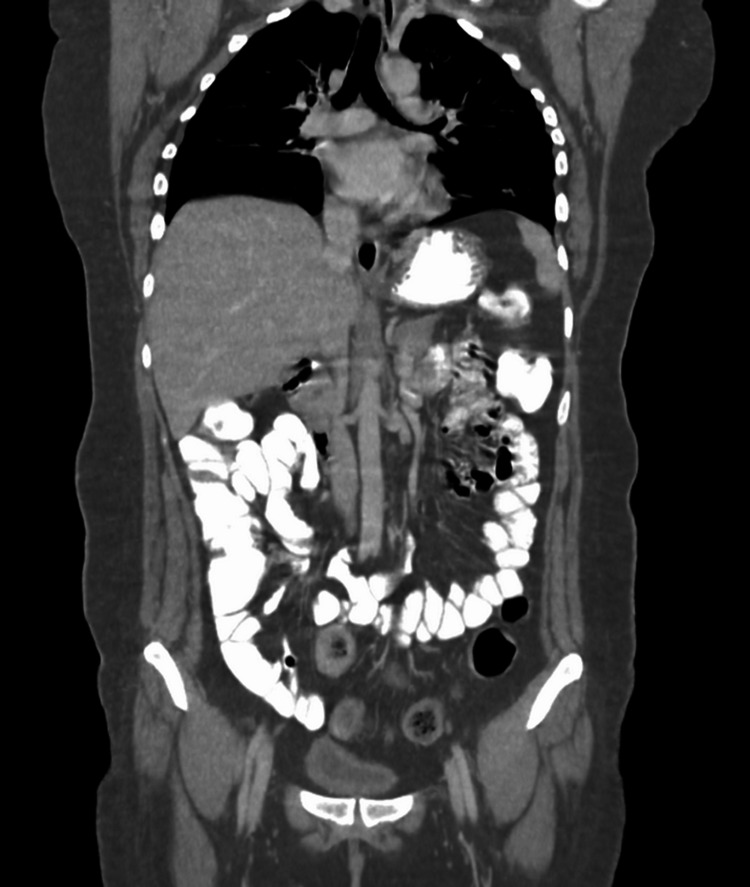
CT scan image of thorax, abdomen, and pelvis with contrast showing no significant abnormalities in soft tissues and organs.

**Figure 4:**
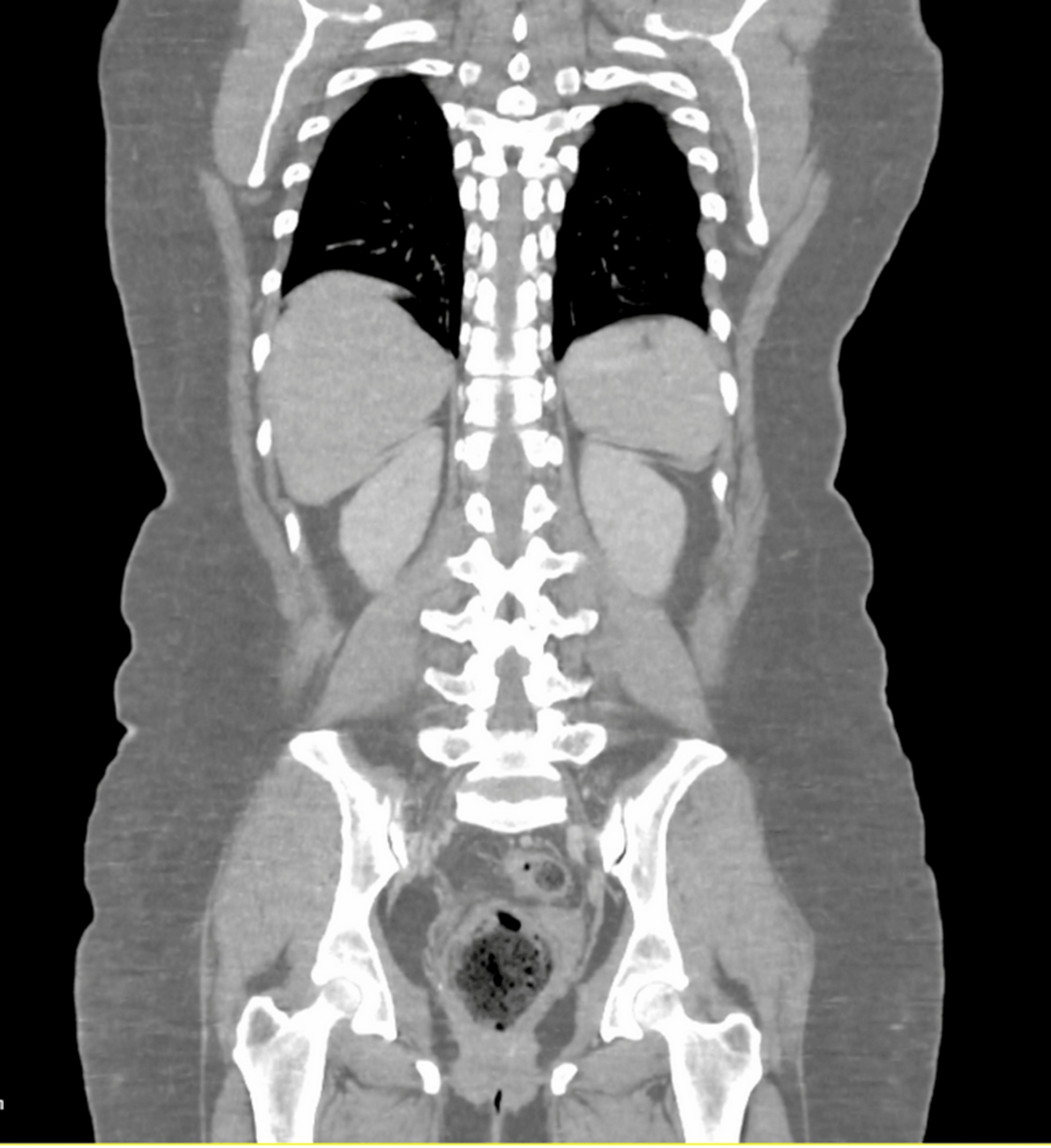
CT scan image of thorax, abdomen, and pelvis with contrast showing no significant abnormalities in bone structure.

Additional investigations were conducted to identify a potential etiology and exclude malignancy. Brain CT scans with and without contrast revealed no acute bleeding or any other lesions. An abdominal ultrasound indicated mild fatty liver, but CT scans of the neck, thorax, abdomen, and pelvis with contrast showed no evidence of neoplastic processes (Figures 3, 4).

**Figure 5:**
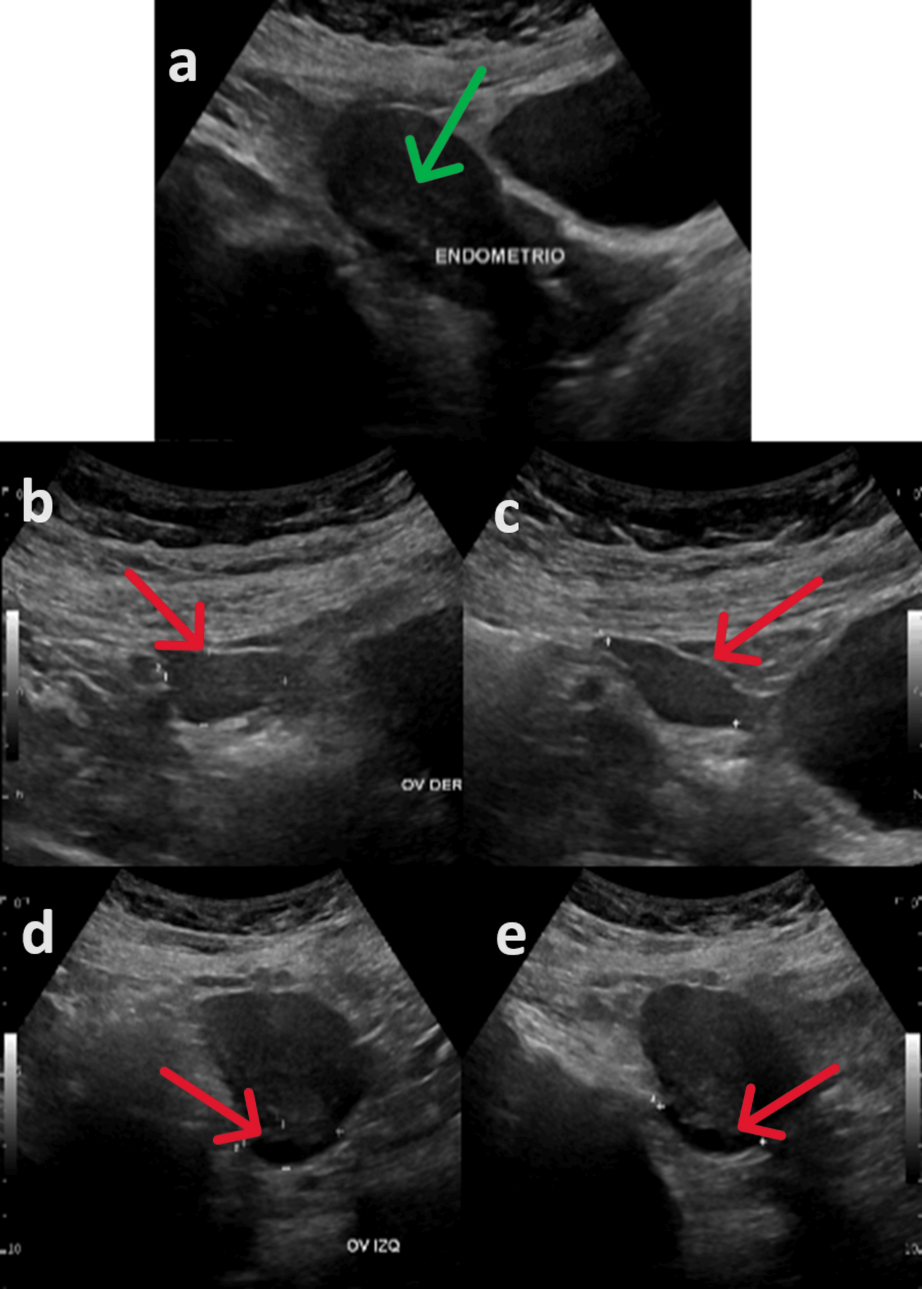
Gynecologic ultrasound showing uterus, right and left ovaries with no significant abnormalities. The images show the uterus, with green arrow pointing to the endometrium (a), the right ovary (b, c), and the left ovary, with red arrows pointing towards the ovary (d, e).

A breast ultrasound identified benign lesions classified as Breast Imaging-Reporting and Data System 2 (BI-RADS 2), and a gynecological ultrasound was normal (Figures 5a-5e). Esophagogastroduodenoscopy revealed chronic atrophic gastritis, while colonoscopy results were entirely normal.

Based on the clinical presentation of opsoclonus, myoclonus, and ataxia, along with dizziness, gait instability, memory loss, and sleep disturbances the patient was managed as an opsoclonus-myoclonus-ataxia syndrome. The patient received IV methylprednisolone 1 g/day for five days, five sessions of plasmapheresis, and IV immunoglobulin 55 g/day for five days with significant but partial improvement of the symptoms. The patient has been monitored over the past year, and no tumors have been detected.

## Discussion

OMAS is an extremely rare disorder that primarily affects children, with a notably low incidence in the adult population [4]. The classic clinical presentation of OMAS typically includes a triad of opsoclonus, myoclonus, and ataxia. However, most patients present with less specific symptoms, such as nausea, vomiting, fatigue, dizziness, balance issues, and disturbances in behavior and sleep, as the case presented [1,5]. While opsoclonus and myoclonus are common features, they may be absent or appear with a delayed onset, leading to potential misdiagnoses based solely on the presence of ataxia [1,5,6]. 

The diagnosis of OMAS is frequently delayed due to its rarity, the limited clinical awareness among neurologists, the intermittent nature of opsoclonus, and the common underestimation of accompanying symptoms [7].

Opsoclonus is a key element in the differential diagnosis of patients with OMAS. It is characterized by involuntary, random, and repetitive high-frequency saccadic eye movements (10-25 Hz) that are irregular in amplitude and occur in all directions without intersaccadic intervals [3]. These movements intensify with fixation or random eye movements and persist even during sleep or when the eyelids are closed [1].

The underlying mechanisms of opsoclonus are still not well understood. It is believed that burst neurons in the paramedian pontine reticular formation and the rostral interstitial nucleus of Cajal are responsible for generating saccadic eye movements. Normally, omnipause cells in the pontine nucleus raphe interpositus inhibit these burst neurons to prevent unwanted saccades [1]. Damage to these omnipause cells is one proposed mechanism for opsoclonus; however, neuropathological evidence supporting this theory is lacking [8]. Another hypothesis, the brainstem theory, suggests that saccadic oscillations result from changes in the membrane properties of saccadic burst neurons [5]. Alternatively, the cerebellar theory proposes that dysfunctional Purkinje cells in the dorsal vermis fail to inhibit the fastigial nuclei in the cerebellum, which in turn disrupts the inhibition of omnipause neurons [3].

The pathophysiology of OMAS remains unclear, but there is a presumption that it is an immune-mediated condition caused by cross-reacting antibodies [7]. In adults, the most common underlying causes include paraneoplastic, parainfectious, toxic-metabolic, and idiopathic factors, making an extensive diagnostic workup essential for every patient presenting with this condition [5]. However, in many instances, no definitive cause is identified [3]. Reported parainfectious cases of OMAS have been linked to various pathogens, including HIV, *Mycoplasma pneumoniae*, *Salmonella enterica*, Hepatitis C virus, rotavirus, chickenpox, and mumps [1].

Paraneoplastic cases are commonly associated with small-cell lung cancer, breast cancer, and ovarian cancer [3]. In paraneoplastic opsoclonus, several autoantibodies have been identified, including anti-Ri (ANNA-2), anti-Yo (PCA-1), anti-Hu (ANNA-1), anti-Ma1, anti-Ma2, anti-amphiphysin, anti-CRMP-5/anti-CV2, anti-Zic2, and anti-neurofilaments. Despite the presence of various autoantibodies in some cases, most patients with opsoclonus are seronegative for all known antineuronal antibodies [3,5]. It is very important to rule out malignancies due to the high incidence of underlying tumors and partial symptom relief through tumor excision. To rule out underlying tumors, all patients should undergo CT scans of the chest, abdomen, and pelvis, as well as brain MRI and positron emission tomography (PET). In our case, PET was unavailable; however, the patient has been monitored with serial imaging studies to detect any malignancies. Literature suggests that if initial imaging results are negative, follow-up evaluations should be repeated every three months [1]. 

In the case presented, all infectious and neoplastic causes were excluded, leading to a diagnosis of idiopathic OMAS. It is crucial to note that even when diagnostic workups are negative, idiopathic OMAS may still be triggered by a viral infection [3]. Once the diagnosis is confirmed, the management of OMAS is based on corticosteroids or IVIG regardless of the tumor status [1]. In the case presented here, the patient responded well to the treatment.

## Conclusions

This report describes a case of adult-onset idiopathic opsoclonus-myoclonus-ataxia syndrome, which is rarely reported in Central America. Although the exact pathophysiology of OMAS remains uncertain, evidence suggests it has an autoimmune mechanism. The diagnosis should be focused on ruling out underlying neoplasia and infections, and the mainstay of management is immunosuppressive therapy.
